# Implant survival in patients with oral cancer: A 5-year follow-up

**DOI:** 10.4317/jced.54937

**Published:** 2018-06-01

**Authors:** Rafael Flores-Ruiz, Lizett Castellanos-Cosano, María-Angeles Serrera-Figallo, Eloy Cano-Díaz, Daniel Torres-Lagares, José-Luis Gutiérrez-Pérez

**Affiliations:** 1 Department of Stomatology, School of Dentistry, University of Seville; 2 Head of the Maxillofacial Department, University Hospital Virgen del Rocío, Seville, Spain

## Abstract

**Background:**

To evaluate the evolution of patients rehabilitated with endosseous implants after oral cancer treatment.

**Material and Methods:**

An observational retrospective study was carried out between 1991 and 2011 with a sample consisting of patients with oral cancer who had been referred for rehabilitation to the Prosthetics Rehabilitation Unit from the Oral and Maxillofacial Surgery Unit of the Virgen del Rocío University Hospital. All these patients have overcome oral cancer, and have a five-year follow-up after their oral rehabilitation. Age, sex, smoking habits, oral pathology, type of treatment of oral pathology, edentulism, receptor bone, prosthetic rehabilitation, timeouts, working time and evolution were studied. SPSS Statistics was used for statistical analysis of the variables studied. A chi-square test centered on the survival rate of implants placed in relation to the other recorded variables was performed.

**Results:**

17 patients were treated for cancer and rehabilitated with implant prosthetics, with a total of 106 implants placed. 32% were partially edentulous patients (4 patients), and 68.2% were completely edentulous patients (13 patients). An implant survival rate of 87.7% was observed at 5 years. In the upper maxilla, the survival rate was 79.2%, and in the mandible 93.7% (*p* = 0.03). 91 implants were placed in native receptor bones (85.8%), with only 15 implants placed in grafted receptor bone (14.2%). According to the type of receptor bone, in native receptor bones, 9 implants failed (90.1% of implant survival), while in grafted receptor bones, 4 implants failed (overall 73.3% implant survival rate) (*p* = 0.08).

**Conclusions:**

Although a high survival rate was obtained in this study (with lower survival rates seen in mandible and grafted bone), prospective long-term studies are needed to assess the relationship between radiotherapy doses and the time required for implant placement, prosthetic protocol used, and type of implants used in patients selected for prosthetic rehabilitation.

** Key words:**Dental implant, oral cancer, survival.

## Introduction

Implantology has led to significant changes in the planning of prosthetic rehabilitation, mainly in patients who have suffered anatomical alterations after surgical treatment of cancer (1-3). Implants in fully edentulous patients help to maintain bone volume, stomatognathic function, masticatory muscle activity and aesthetics, all while positively impacting patient psychological health and quality of life (4,5). In the case of partial edentulism, the use of endosseous implants in the support of prostheses against the teeth usually improves stability and retention of the prosthesis (6).

Although prosthetic rehabilitation has clear benefits for patient quality of life, it is not a systematic treatment commonly used in oncology patients. Patient medical history and the prognosis should be thoroughly evaluated. Likewise, the patient must show a willingness to receive implant rehabilitative treatment, as they must commit to maintaining it (7,8).

The presence of cancer recurrence more than two years after the treatment of the underlying pathology is less than 5% (9), so it seems reasonable for more advanced treatments to be performed only when the patient has surpassed this high-risk period. The patient’s overall situation should be evaluated, and waiting time should be established according to their risk for osseointegration of the implants (10).

Fixed-implant prostheses are more aesthetic and are aimed at increasing patient satisfaction, although they do not always achieve this. The requirements for this type of prosthesis are very high, and due to previous surgeries performed on patients with cancer, in addition to being more difficult to place and attain adequate emergency implants, they involve greater complexity of occlusal stability and more difficult maintenance and revision of the less accessible area (11).

The placement of implant-assisted rehabilitation prostheses in patients with cancer has shown a high degree of satisfaction and there are numerous advantages: they require a smaller number of implants, facilitate oral hygiene, make it easier to obtain greater occlusal stability and good distribution of occlusal forces, and are a less expensive treatment. In turn, these prostheses help restore deficiencies in white and hard tissue without need for additional surgeries (12).

The objective of this study was to evaluate the evolution of patients rehabilitated with endosseous implants after treatment of oral pathology (primarily oral cancer) referred to the Unit of Prosthetic Rehabilitation of the University Hospital Virgen del Rocío between 1991-2011.

## Material and Methods

An observational retrospective descriptive study of patients rehabilitated with endosseous implants after oral cancer treatment during the years 1991–2011 was carried out, analyzing the type, characteristics, treatment and follow-up of the oral rehabilitation methodology implemented. The follow-up period was 5 years.

The inclusion criteria were patients with malignant neoplasia who after their treatment had been referred to the Unit of Prosthetic Rehabilitation of the Oral and Maxillofacial Surgery Unit of the University Hospital Virgen del Rocío for rehabilitation with a five-year follow-up period. Exclusion criteria were patients previously rehabilitated by implant prostheses, patients for whom rehabilitation treatment was contraindicated, and patients from whom information regarding the variables studied could not be obtained.

Information from patients included in the study obtained from the Hospital Clinical Histories of the Prosthetic Rehabilitation Unit was entered into a data collection sheet. The variables studied prior to treatment, during prosthetic treatment and after treatment were all recorded. These variables were age, sex, smoking habits, oral pathology, type of treatment of the oral pathology, edentulism, implant receptor bone, prosthetic rehabilitation, timeouts, working time and implant survival rate after 5 years.

Smoking habits were classed into 1 of 4 categories: 0–10 cigarettes per day, 10–20 cigarettes per day, 20-30 cigarettes per day and more than 30 cigarettes per day.

The Alcohol Use Disorders Identification Test (AUDIT) was used to assess patients’ alcohol consumption (13). Risk categories in the typical consumption table were: low-risk drinkers, ≤ 17 SDU (170 g) for females and ≤ 28 SDU (280 g) for males per week; moderate-risk drinkers, > 17 SDU for females and > 28 SDU for males per week; and high-risk drinkers, > 28 SDU for females and > 42 SDU for males per week (14).

We have defined (and recorded) implant survival as when an implant is successfully kept in the mouth and performing its function. The present study was approved by the Ethics Committee of the University of Seville, and patients provided consent for their clinical data to be used for scientific purposes without being identified.

SPSS Statistics was used to analyze the variables studied. A chi-square test (for the study of the distribution of the different variables in the sample) was also carried out.

## Results

A total of 17 patients were treated with oncological pathology and rehabilitated with implant prostheses, with a total of 106 implants placed. The pre-prosthetic variables studied in patients are shown by age in  Table 1, while the post-surgical variables are found in  Table 2 and  Table 3.

**Table 1:**
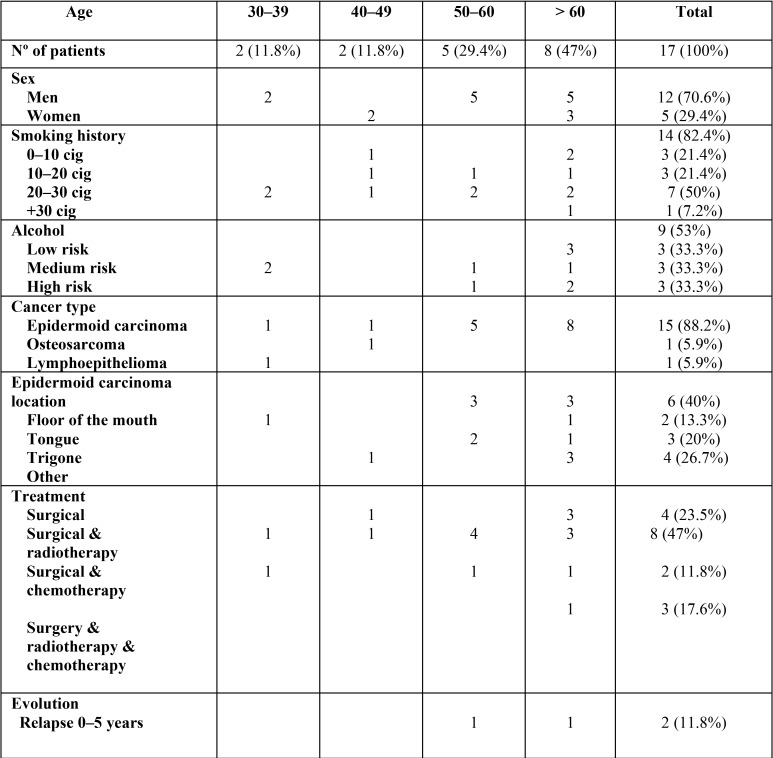
Patient pre-prosthetic variables by age.

**Table 2:**
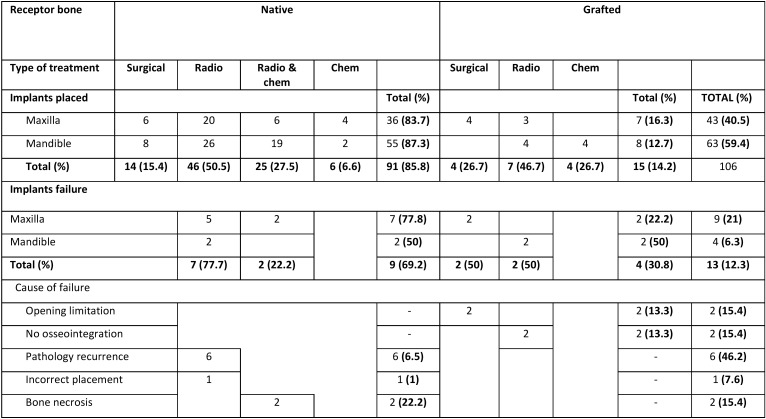
Implant survival variables.

**Table 3:**
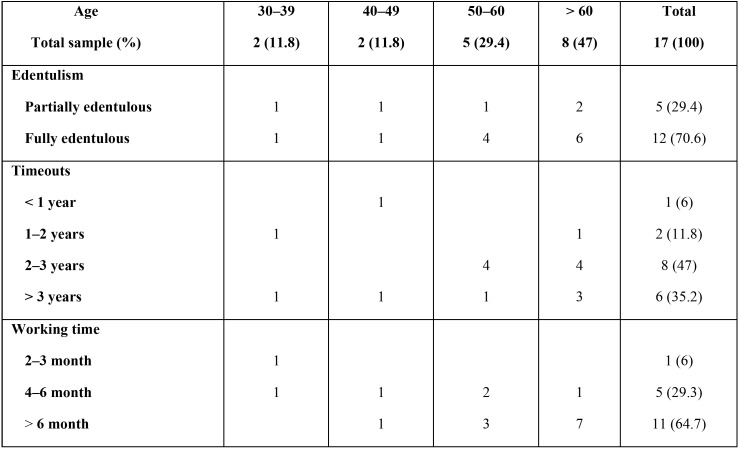
Post-surgical variables of rehabilitated patients by age.

Sample characteristics 

Regarding age, 3 patients (11.8%) fell under the 30–39 to 40–49 year age group, 5 patients between 50–60 years old (29.4%), and 8 patients older than 60 (47%). 29.4% (5 patients) of the sample were women, and 70.6% were men (12 patients). 14 patients (82.4%) were smokers and 3 (17.6%) were non-smokers. The total number of patients who consumed alcohol was 9 (53%), with prudent drinkers (3 patients), moderate drinkers (3 patients) and excessive drinkers (3 patients) each making up 33.3% of this population. None of the evaluated patients continued smoking or drinking after pathology diagnosis.

Of the total sample, 15 patients presented with epidermoid carcinoma (88.2%), one patient with osteosarcoma (5.9%), and one patient with cavum lymphoepithelioma (5.9%). The most frequent localization for squamous cell carcinoma was in the floor of the mouth (40%), retromolar trigone (20%) and tongue (13.3%) ( Table 1). The most frequent modality used to treat patients was surgery with postoperative radiotherapy (47%), followed by surgery as the only treatment for that particular case alone (23.5%) and only in that case, with 17.6% of the cases being performed after surgery with a combination of radiotherapy and chemotherapy ( Table 1).

Implant and treatment characteristics

A total of 106 implants were placed. Data on the type of bone and the type of cancer treatment performed can be found in  Table 2. Of the 17 patients rehabilitated, 29% were partially edentulous patients (5 patients) and 71% were fully edentulous (12 patients), type of edentulism, timeouts and working time by age could be seen in  Table 3. In 17 patients, 19 prosthetic rehabilitations were performed on implants: 10 overdentures (52.6%); 7 hybrid-type fixed prostheses (36.8%) and 2 metal-ceramic fixed prostheses (10.6%).

Of the 106 total implants placed, a total of 43 implants (40.5%) were placed in the upper maxillary bone, with 63 implants (59.4%) placed in the lower jaw. 91 implants (85.8%) were placed in native receptor bone, of which 36 were placed in the maxilla (83.7%) and 55 in the mandible (87.3%) ( Table 2). Only 15 implants (14.2%) were placed in grafted receptor bone, of which 7 implants were placed in the upper jaw (16.3) and 8 implants were placed in the lower jaw (12.7%) ( Table 2).

Post-rehabilitation follow-up

All patients had a minimum follow-up period of 5 years, during which period only 2 patients in the sample studied presented recurrence of tumoral pathology (9%), being more frequent in patients over the age of 50 (100%).

An implant survival rate of 87.7% was found after 5 years of oncological treatment. Of the total number of implants placed, 13 implants failed (12.3%), of which 9 implants were placed in the maxilla (79.2% implant survival rate) and 4 implants in the lower jaw (93.7% implant survival rate) (*p* = 0.03). Regarding the type of receptor bone, 9 failed implants had been placed in native receptor bone (90.1% implant survival) and 4 failed implants in grafted receptor bone (73.3% implant survival) (*p* = 0.08;  Table 2).

## Discussion

Prosthetic rehabilitative treatment with or without dental implants leads to an improvement in patient’s quality of life through functionality, aesthetics and social rehabilitation (5).

Chiapasco *et al.* (15) presented the exclusion criteria for treatment with implants: a) patients with a poor prognosis or whose health is compromised systemically; B) patients who have undergone resection of the posterior part of the jaw or mandible with sufficient remaining dentition to ensure acceptable chewing; C) patients with recurring oral carcinoma who continue to consume alcohol or tobacco; and d) non-cooperative patients.

Long-term data collection of patients with oral tumors is difficult as their life expectancy is often reduced. One study found that 50% of patients with oral tumors died within 2.3 years of ending treatment, i.e. before reaching 5 years of survival (16). In this case, all patients survived after 5 year of oncological treatment, although 2 of them had experienced a relapse during this period, and as a result 6 dental implants had to be removed.

Smoking and regular alcohol consumption are often associated with the development of oral tumors, increasing this risk from 6 to 15. After quitting, this risk decreases and may disappear within 5-10 years (17). In this sample, 59% of patients smoked, data very close to revealed shown by Katsoulis (18) in a study in 2013 with 54%. Squamous cell carcinoma was observed in 88% of this patients, a percentage higher than that observed by other authors with 78% (18).

Timeouts

The ideal time interval for radiotherapy treatment so as not to affect osseointegration of implants is a matter of some debate (19). Some authors advocate immediate placement after ablative surgery (during the same surgical procedure) (20). The advantages of this procedure are that the implant will have a better osseointegration before radiotherapy, only one procedure is needed, and speech, mastication or aesthetics are either unaltered or can be treated with hyperbaric oxygen. The main disadvantages include added risk for the patient, as radiotherapy treatment must be delayed, possible postoperative complications respective surgery, and even recurrence of the underlying pathology.

Authors such as Hancock (21) and Brogniez (7) suggest that a waiting period of 6 months may be sufficient. Accordingly, Vish *et al.* (22) conclude that six months after completion of radiotherapy, the success rate for implant survival is not affected. The difference in survival percentages between implants inserted <1 year and ≥ 1 year after irradiation is not significant (76% and 81%, respectively).

Other studies (23,24) prefer a minimum waiting period of 24 months, since this period allows for consolidation of the patient’s health status and can rule out related tumor pathologies and facilitate recovery of the maxilla with adequate vascularization, consolidation of the reconstruction area, restoration of soft tissues affected by the treatment and, not least, the patient’s own psychological state. In this study all implants were deferred. Although some patients in this sample were rehabilitated within two years of completing cancer treatment, the vast majority of patients were rehabilitated after this period (> 70%). An implant survival rate of 87.7% was obtained after 5 years of follow-up, with 79.2% in the upper jaw and 93.7% in the mandible.

More than 2 years were required in some patients for various reasons: they had overcome the illness but they were not mentally prepared to undergo surgery, they did not want to have another surgery at that time, or they could not attend their appointment in the rehabilitation unit. The protocol used in this study considers the restitution of basic functions by the patient to be essential, focusing initially on the restoration of health status after the surgical treatment of oncological pathology, and, once achieved, the subsequent placement of endosseous implants.

Implant survival 

Combination therapy (radiotherapy and chemotherapy) for tumors and follow-up implantation for functional reconstruction are considered necessary in patients with head and neck cancers (25). Although radiation therapy is commonly applied to the cancerous tumor as an important means of therapy, it can provoke changes in the patient’s soft and hard tissues. Implant failure can occur at different times, although it is most likely to occur within the first few months after placement, before the prosthetic phase begins (19,24). In this study, two implants failed due to necrosis of the receptor bone after the patient had been treated with chemotherapy and radiotherapy, although a 12-month period after completion of oncologic treatment was expected (2.2%).

Yerit *et al.* (26) propose that implants be placed in areas with higher quality bone, as surgical trauma on bone of low quality, along with the effects of the radiotherapy treatment, can diminish the capacity of osseointegration or lead to osteoradionecrosis. All of these changes are considered detrimental to implant survival. Bone loss around implants can occur, often the result of contamination by a mechanical or combined infectious agent, but it is rarely associated with the development of a malignant lesion.

In their 2002 study, Visch *et al.* (22) evaluated differences in survival rates according to location of the implants and the radiation intensity of radiotherapy. With respect to implants placed in locations where radiation was given at less than 50 Gy, 19 of 207 implants failed, achieving a survival rate of 84% at 10 years. In locations where radiation was greater than 50 Gy, the survival rate was 71%. Jisander *et al.* (27) observed that most complications occurred at doses greater than 50 Gy. This may be due to the reduction in vascularization that occurs when bone is irradiated at that dose. Anatomical characteristics may also influence implant survival rates in the maxilla and mandible (79.2% vs. 93.7%, *p* = 0.03).

Kanchan *et al.* (28) assessed the rate of osseointegration (ROI) and overall 5-year survival rate (OSR) of implants placed in native and grafted mandibles after ablative surgery, either with or without radiation therapy, in an Indian population. The ROI and OSR for implants was 88% and 77%, respectively. Nasser in 2013 (29) studied the survival of implants in patients who had been irradiated after diagnosis of oral cancer and found that in 34 articles, implants in post-radiation patients showed an average survival rate of 88.9% (over 3,775 implants). These results are similar to those found in this study, in which a survival rate of 87.7% was found at 5 years of follow-up. In this study, the high survival rate may be due to adequate choice of patients and to the rigorous protocol applied in the Oncology Rehabilitation Unit.

## Conclusions

In patients treated for carcinoma in the oral cavity, there is no consensus as to the timeouts needed to achieve successful survival after placement of endosseous implants or for placement of prostheses. Although a high survival rate was obtained in this study (with a slightly lower survival rate observed in implants placed in grafted bone and maxillary bone), the limited sample size makes further prospective long-term studies necessary in order to evaluate the relationship between radiotherapy doses, waiting time required for the placement of the implants, prosthetic protocols followed, and the type of patient selected for prosthetic rehabilitation.
